# Parenteral nutrition at the palliative phase of advanced cancer: the ALIM-K study protocol for a randomized controlled trial

**DOI:** 10.1186/1745-6215-15-370

**Published:** 2014-09-24

**Authors:** Lionel Pazart, Elodie Cretin, Ghislain Grodard, Cecile Cornet, Florence Mathieu-Nicot, Franck Bonnetain, Mariette Mercier, Patrice Cuynet, Carole Bouleuc, Regis Aubry, the ALIM-K study investigational group

**Affiliations:** Inserm CIC 1431, CHRU de Besançon, Besançon, France; Département douleur – Soins palliatifs, CHRU de Besançon, Besançon, France; Espace Ethique Bourgogne Franche-Comté, Franche-Comté, France; Laboratoire EA 3188 de psychologie de Besançon, Université de Franche-Comté, Besançon, France; Département de soins de support et de soins palliatifs, Institut Curie, Paris, France; Diététique, CHRU de Besançon, Besançon, France; Laboratoire Logiques de l’Agir EA 2274, Université de Franche-Comté, Besançon, France; Plateforme « Qualité de vie et cancer », Besançon, France; EA 3181, Université de Franche-Comté, Franche-Comté, France; Inserm CIT 808, University Hospital Besançon, Besançon, 25030 France

**Keywords:** Malnutrition, End-stage cancer, Palliative care, Parenteral nutrition, Randomized controlled trial

## Abstract

**Background:**

Malnutrition is a common complication in patients at the palliative stage of cancer. During the curative phase of cancer, optimal enteral or parenteral nutrition intake can reduce morbidity and mortality, and improve quality of life. When the main goal of treatment becomes palliative, introduction of artificial nutrition is controversial. Although scientific societies do not recommend the introduction of artificial nutrition in all cases of malnutrition, especially in hypophagic patients if their life expectancy is shorter than 2 months, considerable differences in the use of parenteral nutrition in nonsurgical oncology practice are noted around the world. One explanation is a paucity of well-conducted randomized controlled trials in these situations, and consequently, the risk/benefit ratio of parenteral nutrition and its impact on quality of life in palliative care remains uncertain.

**Methods/design:**

The ALIM-K study is a French national multicenter randomized controlled trial designed to evaluate the effectiveness of parenteral nutrition, versus an exclusive oral-feeding supply, on the quality of life of malnourished patients who have a functional digestive tube and who are at the palliative phase of advanced cancer with a life expectancy of more than 2 months.

**Discussion:**

This article presents the methodologic options chosen for our study, and in particular, the choice of the Zelen method of randomization, the definition of the main end point (quality of life), the choice of comparator (oral feeding), and the inclusion criteria (life expectancy of more than 2 months), which are all critical points in building a randomized controlled trial in the setting of palliative care.

**Trial registration:**

This study was registered with the clinical trials database ClinicalTrials.gov on May 27, 2014, under the number NCT02151214.

## Background

Malnutrition in cancer patients is well established as a contributor to morbidity and mortality, and its prevalence remains high, despite considerable progression in cancer therapy over the last 10–year period. According to reports, malnutrition affects 20% to 50% of cancer patients [1], and its consequences in terms of morbidity and mortality are well described [2, 3]. Anticancer treatments compound this situation by the addition of frequent digestive disorders (nausea, vomiting, abdominal cramps, mucitis, paralytic ileus, malabsorption). The sometimes severe vomiting despite adjunctive use of antiemetics or optimization of chemotherapy regimens can also be a possible cause of malnutrition.

In the last few years, malnutrition in cancer has garnered increasing attention, and this is reflected by the publication of specific guidelines and research reports, whose number has increased by 84% in the last 5 years (131 publications) as compared with the previous 5 years (71 publications) (based on a Medline search for the terms “malnutrition” AND “Cancer” limited to “Clinical Trials OR meta-analysis OR guideline” as filters, as performed on August 12, 2014).

Accordingly, the European Society for Parenteral and Enteral Nutrition (ESPEN) has published specific guidelines for the use of artificial nutrition in cancer patients in the nonsurgical context [4, 5]. For patients undergoing curative treatment, management of malnutrition is based on enteral nutrition when the digestive tract is functional, and parenteral nutrition is indicated for patients whose digestive tract is not functional (for example, in patients with peritoneal carcinosis with intestinal occlusion in the context of gynecologic cancers). Parenteral nutrition offers the possibility of increasing or ensuring nutrient intake in patients in whom normal food intake is insufficient and enteral nutrition is either not feasible, contraindicated, or not accepted by the patient [5]. The duration of this type of nutrition can be up to several months. This type of artificial nutrition yields a significant increase in survival, and also a significant gain in terms of disease-related morbidity [4–6]. Serious complications can occur in the short, medium, or long term, and mainly comprise catheter infections, septic complications, hyponatremia, hypokalemia, hypomagnesemia, hypophosphatemia, or hyperglycemia, resulting in dehydration with electrolyte disturbances. These in turn may cause coma, hypertriglyceridemia, or hepatobiliary complications in the longer term, with risk of progression to active fibrosis and cirrhosis.

Considerable controversy remains as to whether artificial nutrition should be introduced at the palliative stage of cancer. The European Society for Parenteral and Enteral Nutrition (ESPEN) has published specific guidelines for the use of artificial nutrition (enteral or parenteral) in end-stage cancer [4, 5]. Despite efforts by scientific societies to be restrictive with the indications for artificial nutrition, wide variations in the use of parenteral nutrition in nonsurgical oncology practice exist around the world. Withdrawing or withholding artificial nutrition in palliative care is often interpreted by the patient, their family, and/or the caregivers as an abandonment that can often lead to ethical dilemmas among health care providers [7]. Health care providers seem to lack information [7] and consensus regarding the introduction or withdrawal of artificial nutrition at the end of life, and most existing guidance stems from expert agreement, with a low level of evidence [8].

In case of end-stage cancer, the results are even less salient, and to date, few clinical studies have evaluated the risk-benefit ratio of artificial nutrition in the context of palliative care. Agreement exists among all healthcare professionals that it is not advisable or desirable to introduce artificial nutrition in an aphagic or hypophagic patient at the end stage of cancer, if their life expectancy is shorter than the expected duration that it would take to die of starvation (that is, less than 2 months) [8, 9]. Conversely, it is unclear what should be done for such patients with incurable cancer when life expectancy is longer than 2 months.

A major source of controversy in daily practice arises from the involvement of several different specialists, who each focus on different outcomes. For example, the oncologist focuses on reducing tumor growth and improving survival, whereas the nutritionist focuses on balancing food intake and biologic parameters, whereas supportive carers are primarily concerned with the well-being and quality of life of the patient. Unfortunately, it is not uncommon for oncologists to persist with their treatment objectives, even when some consider that artificial nutrition could accelerate tumor growth. Nutritionists and dietitians cite the benefits of a balanced diet in responding to energy needs, thus helping the patient to feel better.

Last, palliative or supportive caregivers are reluctant to administer parenteral feeding, for fear of a futile and risky intervention, which could lead to serious complications and loss of autonomy with unnecessary prolongation of the patient’s suffering and end-of-life process, rather than extending survival with an acceptable quality of life.

One of the main objectives of palliative care is the improvement of, or failing that, maximizing delay in the deterioration of health-related quality of life. In this context, the expected favorable effects are counterbalanced by the constraints and complications related to artificial nutrition, particularly when oral feeding is still possible. Thus, artificial nutrition is not recommended in patients if the nutritional status, and/or calorie intake, remain satisfactory [8, 9].

A recent nationwide cross-sectional study performed by the research network in palliative care in Sweden (PANIS) described off-label use. The authors studied 1,083 patients with malignant tumors at the palliative stage, and noted that 13% were receiving artificial nutrition [10]. The data showed that parenteral nutrition was the most frequent type used (in two thirds of cases), with wide regional variations. Other countries have reported almost systematic use of artificial nutrition in this context (80% of patients at admission to the Seoul main hospital, and up to 83% at 2 days before death) [11]. Weight loss and loss of appetite were the main reasons justifying initiation of artificial nutrition, despite the possibility of oral feeding. Initiating artificial nutrition at this stage of disease seems to be less guided by scientific evidence, and more by subjective motivations linked to beliefs, cultural or religious traditions, the symbolism of the function of eating, the deep-seated fear of dying of hunger, and the vision of care.

Parenteral nutrition could contribute to maintaining or improving autonomy, thanks to increased muscle mass, reduced feelings of asthenia, and overall improvement in general well-being. Only one randomized controlled trial to date has shown that parenteral nutrition prolongs overall survival of cachexic patients, as compared with oral feeding alone, associated in both arms of the study with cyclooxygenase, insulin, and erythropoietin treatment [12]. The risk-benefit ratio of parenteral nutrition, particularly in terms of health-related quality of life, is thus poorly documented among patients with malnutrition but with a functional digestive tract during the end stages of cancer.

Therefore, we initiated a randomized, controlled trial to evaluate the clinical benefit, as assessed by patients’ health-related quality of life, of supplemental parenteral nutrition versus no parenteral nutrition, in the context of palliative care at the end stage of cancer.

### Hypotheses and objectives

In view of the uncertainty regarding the risk/benefit ratio of supplemental parenteral nutrition, we aim to investigate which of two management options (initiation of supplemental parenteral nutrition or no introduction of parenteral nutrition) proves to be superior in terms of health-related quality of life without a significant decrease in survival, in the palliative phase of cancer.

The primary objective of the ALIM-K study is to evaluate the clinical benefit in terms of health-related quality of life, as evaluated at 2 months (or at treatment discontinuation for patients who discontinue treatment allocation before 2 months), by using the European Organisation for Research and Treatment of Cancer (EORTC) Quality of Life Questionnaire Core 15 Palliative (QLQ-C15-PAL), and in particular, the physical function, global health, and fatigue subscales [13], of the initiation of supplemental parenteral nutrition as compared with no parenteral nutrition, in malnourished cancer patients at the palliative stage who have a functional digestive tract, and in the absence of any curative therapy.

Secondary objectives are to compare the effects of both types of nutrition on overall survival and nutritional parameters.

### Trial design

We initiated a multicenter, prospective, 1:1 randomized, controlled, parallel-group trial. The experimental arm consists of initiation of supplemental parenteral nutrition. The control arm consists of pursuing oral feeding alone.

To minimize the difficulty of obtaining informed consent and to stimulate accrual, we used the Zelen single-consent design [14] to randomize patients before informed consent has been obtained. After randomization has been performed, only patients allocated to the intervention group are approached and asked to consent to the study intervention (parenteral nutrition in this case). According to Zelen, patients allocated to the control group and receiving the standard of care (oral feeding in this case) do not need to be informed because these patients are simply going to receive the usual therapy, and therefore, do not constitute a “study population”. Patients allocated to the intervention group who refuse consent are reassigned to the control group.

## Methods/design

### Participants, interventions, and outcomes

#### Study setting

This study will be conducted nationwide in France in nine comprehensive cancer centers selected for their expertise, and qualified to treat patients with more-advanced cancers, as well as in two university teaching hospitals (University Hospital of Besançon and Henri Mondor Hospital, Creteil, France).

#### *Inclusion and exclusion criteria (Table* 1*)*

**Table 1:** **Inclusion and exclusion criteria for participants**

Inclusion criteria	Exclusion criteria
Adult patients (aged >18 years) with cancer at the palliative stage	Any disorder preventing oral ingestion (cancer of the upper aerodigestive tract, esophagus, or stomach)
Specific curative treatment discontinued or still ongoing but with little expected benefit in terms of overall survival	Nonfunctional digestive tract
Life expectancy greater than 2 months	Symptomatic peritoneal carcinosis
Malnutrition defined as Body Mass Index <18.5 kg/m^2^ if age <70 years, <21 kg/m^2^ if age ≥70 years; or weight loss of 2% in 1 week, or 5% in 1 month, or 10% in 6 months	Hematologic malignancies
Patient no longer experiences a sensation of hunger	Myocardial infarction or stroke in the previous 6 months
Patient has lost appetite	Acute renal insufficiency (creatinine clearance < 30 ml/min) or heart failure (left ventricular ejection fraction <30%)
Patient has reduced food intake	Parenteral nutrition ongoing or dating from <1 month
Functional central venous catheter in place	Presence of gastrostomy or jejunostomy
Functional digestive tract	Persisting sensation of hunger
Patient able to understand and speak French	
	Inability to complete quality-of-life questionnaires (due to psychiatric disorders, attention disorders, or cognitive disorders).
	Patients participating in another ongoing trial
	Adult patients under legal guardianship

Inclusion criteria are as follows: adult patients (aged >18 years) with cancer at the palliative stage (that is, patients in whom the main aim of treatment is to limit pain and discomfort). In these patients, curative treatment either has been discontinued, or may still be ongoing but with little expected benefit in terms of overall survival. Life expectancy must be longer than 2 months. To be eligible, patients must have a functional digestive tract and present malnutrition [15–17], defined as a body mass index (BMI) <18.5 kg/m^2^ in patients aged <70 years or <21 kg/m^2^ in patients aged ≥70 years; or weight loss of 2% in 1 week, 5% in 1 month, or 10% in 6 months. Patients with antalgic radiotherapy or scheduled to undergo palliative surgery may be included. Patients must already have a functional central venous catheter in place.

Exclusion criteria are as follows: nonfunctional digestive tract (intestinal occlusion, tumor compression, subocclusive peritoneal carcinosis); any disorder preventing oral ingestion (cancer of the upper aerodigestive tract, esophagus, or stomach); parenteral nutrition that is ongoing or dating from less than 1 month; intravenous chemotherapy through a pump lasting >48 hours, as this is incompatible with administration of parenteral nutritional through the central venous line; presence of gastrostomy or jejunostomy; or persisting sensation of hunger in aphagic patients. We will also exclude patients with hematologic cancers undergoing bone marrow transplant, patients with acute renal failure (defined as creatinine clearance <30 ml/min) or heart failure (defined as a left ventricular ejection fraction <30%); adult patients under legal guardianship or those unable to respond to the quality-of-life questionnaire (due to psychiatric disorders, attention disorders, or cognitive disorders). Patients participating in another ongoing clinical trial will also be excluded.

#### Intervention in each group

Two groups will be constituted by random allocation. In the intervention group (“parenteral nutrition” group), parenteral nutrition will be initiated, whereas in the control group (“oral feeding” group), nutrition will be exclusively by the oral route, without parenteral nutrition.

At inclusion, patients in both groups will be evaluated by a dietitian with the aim of allowing all patients, regardless of the treatment group to which they are allocated, to pursue oral feeding where possible. Initial evaluation will record the following data:

- ➢ Anthropometric data (weight, ideal weight, height, BMI)
- ➢ Dietetic advice given (previously or during evaluation) regarding uncomfortable symptoms
- ➢ Estimation of theoretic energy requirements in kcal [18] adjusted by a correction factor for the cancer
- ➢ Estimation of theoretic protein requirements (1.2 to 1.5 g of protein per kilogram (kg) per day) [19]
- ➢ Quantitative estimation of daily intake in terms of calories and protein (over 24 hours, as reported by the patient)
- ➢ Visual or verbal analog scale of calorie and protein intake (measured on a scale from 0 to 10)
- ➢ Number of eating intakes per day
- ➢ Oral nutritive supplements: type, dose, and protein-energy content.

The dietitian’s main tasks will be as follows:

- ➢ To provide appropriate dietetic advice aimed at spacing out and enriching food intake
- ➢ To provide specific advice on the management of symptoms related to food intake, such as anorexia, dry mouth, early satiety, dysgeusia, nausea, vomiting, chewing/swallowing disorders, transit disorders
- ➢ To propose, where necessary, prescription of oral nutritional supplements.

In the “oral feeding” group, patients will pursue exclusive oral feeding as usual, tailored to the patient’s needs by a dietitian or nutritionist, according to local practice in each center.

In the “parenteral nutrition” group, parenteral nutrition will be initiated in accordance with current national standards [20], which recommend implementation of parenteral nutrition through a central intravenous line (because the expected duration of use is longer than 2 weeks), to limit the risk of chemically induced phlebitis and to preserve peripheral venous capital. In the context of cancer, the prior presence of an implantable chamber for specific chemotherapy will facilitate administration of parenteral nutrition. Parenteral nutrition through a central line makes it possible to use hyperosmolar mixtures that, thanks to the high rate of flow of the vena cava, do not present significant venous toxicity. The dispensing conditions (continuous or cyclic) and recommended products are detailed later.

**Type of product** Pharmaceutical ternary mixtures will be used, especially those with electrolytes, to limit at-risk handling. No specific brand or product is recommended for the study; each participating center is free to administer parenteral nutrition in accordance with their local practice. The type or brand of product used will be taken into account in the analysis at the end of the study. Micronutrients will be systematically added to the mixture daily via a vial of multivitamins and oligoelements. The addition of electrolytes (sodium, potassium) will be adapted to the patient’s requirements, and added to the mixture if no associated infusion is present. Vitamin K supplementation will also be adapted to the patient’s requirements (by evaluation of vitamin K-dependent factors). Finally, magnesium and phosphorus supplementation will be provided as needed.

**Energy intake** The exact modalities of parenteral nutrition will be left at the physician’s discretion, with the possible help of a dietitian. The recommended dose for an aphagic patient will be 25 to 35 Kcal/kg/day, with 1.2 to 1.5 g/kg/day proteins. Daily intake must not exceed 1.25 times the resting energy expenditure, as calculated by using the Harris-Benedict equation. Energy intake will be adapted according to residual oral food intake, with at least 1,000 Kcal/day and 6 g of nitrogen, on 5 days of 7.

**Administration and follow-up** Parenteral nutrition will be administered via a central venous line (venous catheter or implanted port). The flow rate of the intravenous infusion by the central line must be regular, controlled with a pump (±5%). Flow rate for infusion of carbohydrates must not exceed 4 mg/kg/min; the daily dose of lipids will be <1 g/kg/day. Cyclic administration (over 12 hours at night) is now considered standard practice because of the numerous advantages of this technique, including greater autonomy for the patient, possibility to maintain physical activity, preservation of the physiological alternation between fasting and food intake, the frequent association with oral feeding, and better hepatic tolerance. Patients may be managed in the hospital or at home.

#### Explanation of the choice of comparators

In the context of palliative care for advanced cancer in patients with a functional digestive tract, current national guidelines in France do not recommend artificial nutrition [8, 9]. Thus, the standard of care in this study will be exclusive oral feeding. Enteral nutrition is generally to be avoided in this context, because of its invasive nature and the low tolerance for a stoma catheter or nasogastric feeding tube in conscious patients, and also because it may appear to be a disproportionate measure in this population with limited vital prognosis.

#### Criteria for discontinuing or modifying allocated interventions

Parenteral nutrition may be discontinued at any time by the investigator or by the patient. Discontinuation is mandatory in case of uncontrolled hydroelectrolyte imbalance, insulin-dependent diabetes with persistent fasting glycemia >2 g/L, triglycerides >3 m*M*, or intra- or extraluminal infection.

If the treating physician of a patient allocated to the “oral feeding” group decides, during follow-up, to initiate enteral or parenteral nutrition (irrespective of the reason, including at the request of the patients or their close relatives), follow-up within the ALIM-K study will continue according to the modalities stipulated for the group to which the patient was initially randomized.

In case of serious adverse events or effects leading to discontinuation of the type of feeding intended for the group to which the patient was initially allocated, the patient will be followed up according to the modalities stipulated for that group, because follow-up corresponds to standard recommended practices.

The investigator may decide at any time to change the patient’s management, whether for reasons related to safety, behavior, or administrative requirements, or for any other reason the investigator judges relevant.

#### Strategies to improve adherence to intervention protocols

In randomization with the Zelen design [14], patients are allocated to the treatment group before being approached for informed consent. Patients allocated to the “oral feeding” group will thus be treated in accordance with standard practices, and the Hawthorne effect should be minimized because the Zelen design obviates the need to inform the patient about the idea of participating in a randomized study. Thus, using the Zelen design reduces the difficulty of obtaining informed consent, and increases the rate of participation in the study, by removing the psychological barriers linked to lack of understanding of the need for random allocation in deciding which therapeutic approach to choose, particularly for a patient at the palliative phase of advanced cancer.

#### Relevant concomitant care and interventions that are permitted or prohibited during the trial

No medication is prohibited in the context of the study. All medications usually prescribed for the management of patients may be administered.

#### Outcomes

**Primary outcome** The primary end point is health-related quality of life specific to the palliative phase of cancer, evaluated at 2 months (or at treatment discontinuation for patients who discontinue treatment allocation before 2 months), by using the European Organisation for Research and Treatment of Cancer (EORTC) Quality of Life Questionnaire Core 15 Palliative (QLQ-C15-PAL), in particular, the physical function, global health, and fatigue subscales [13].

The QLQ-C15-PAL will be proposed to the patient at inclusion in the study, and at every follow-up visit until death. If necessary, help is available for patients to complete the questionnaire (reading out the questions, explaining the different response options, taking any notes). The QLQ-C15-PAL is the short version of the QLQ-C30 adapted for patients in palliative care [13]. The 15 items are divided into two functional subscales (physical function, three items; and emotional function, two items); two symptom subscales (fatigue, two items; and pain, two items); five single items (dyspnea, appetite loss, sleeping disorders, constipation, and nausea/vomiting), and one item evaluating global health status/quality of life. Linear transformation of the item responses yields a score for each subscale (or single items) ranging from 0 to 100. A higher score corresponds to a higher level of global quality of life and functional aspects, and a greater level of symptoms on the symptom scale. The subscales physical function, global health status/quality of life, and fatigue will be evaluated for the primary end point. The minimum clinically significant difference is fixed at 10 points [21].

**Secondary outcomes** Secondary end points are as follows:

- ➢ Overall survival, defined as the time from the date of randomization until death of any cause. Patients who are still alive at the end of the study will be counted at the date of last follow-up.
- ➢ Other (non-primary-end point) domains of the QLQ-C15-PAL questionnaire (pain, emotional function, nausea/vomiting, appetite, dyspnea, constipation, and sleep) at 2 months (or at treatment discontinuation for patients who discontinue allocated treatment before).
- ➢ Longitudinal analysis of all domains of the QLQ-C15-PAL.
- ➢ Nutritional parameters: weight, C-reactive protein (CRP), prealbumin, albuminemia.
- ➢ For patients at the end-of-life stage: the Quality of Life at End of Life (QUAL-E) questionnaire will be administered by a clinical research assistant and completed based on the patient’s responses. This questionnaire has been validated in the English-language version [22], and a cross-cultural validation into the French language has been completed [23]. The French-language version comprises 25 questions (compared with 24 in the English version) plus one global question, covering five domains:

- life completion
- relationships with the health care system
- preparation/anticipatory concerns
- symptom impact
- connectedness and affective social support

The five possible response modalities for each item are coded from 1 to 5. The score for each domain is calculated by summing the responses for each item. The global QUAL-E score is the sum of all 26 items. Linear transformation yields score values ranging from 0 (lowest quality of life) to 100 (highest quality of life). The scores from the individual domains of the QUAL-E will also be analyzed.

In the “parenteral nutrition” group, the following information will be recorded:

- ➢ Quantity and duration of treatment actually administered.
- ➢ Biologic results from blood samples taken more than 4 hours after the end of infusion of the last bag of mixture, including blood gases, evaluation of phosphorus, magnesium and calcium levels, hepatic enzymes and triglycerides, and evaluation of glycosuria and ketonuria.
- ➢ Any and all adverse effects.

#### *Time schedule for participants (see Table* 2*)*

**Table 2:** **A schematic diagram of time schedule for enrolment, interventions, assessments, and visits for participants**

	Pre-randomization (day -7 to day 0): Check inclusion and exclusion criteria					
	Zelen's randomization					
	Parenteral nutrition group			Oral feeding group		
	Present "parenteral nutrition" info			Present "oral feeding" info		
	Day 1	Days 2-60*	Day 61-death**	Day 1	Days 2-60*	Day 61-death**
Obtain informed consent	X			X		
Record demographic data	X			X		
Take medical history	X			X		
*Treatment*						
Concomitant treatments	X	X		X	X	
Specific treatment (prior or ongoing)	X	X		X	X	
*Patient status*						
WHO Status	X	X	X	X	X	X
Karnofsky index	X	X		X	X	
Estimated life expectancy	X			X		
*Anthropometric data*						
Height	X			X		
Weight	X	X	X	X	X	X
*Biology*						
Lactic acid dehydrogenase	X			X		
C-reactive protein, albumin, prealbumin	X	X		X	X	
Blood count, electrolytes (including phosphorus, calcium, magnesium)	X	X				
Hepatic enzymes (AST, ALT, PAL, Gamma GT, Bilirubin)	X	X				
Triglycerides	X	X				
Ketonuria, glycosuria	X	X				
Quality of life questionnaires (QLQ-C15-PAL and QUAL-E***)	X	X	X	X	X	X
Socio-economic variables	X			X		
Dietetics consultation	X			X		
Ingesta questionnaire		X	X		X	X
Evaluation of metastatic disease		X			X	
Intervention supportive care		X			X	

*Follow-up consultation at least once per month **Evaluation once per month. ***Administer the QUAL-E if the patients consider themselves to be at the end of life (definition at the discretion of the physician and patient).

*Day -7 to day 0:* Patients who meet the inclusion criteria will be identified from medical files or from regular staff meetings or multidisciplinary case reviews, by the oncologist or the treating physician from palliative care. Once identified, eligible patients will be randomized through a dedicated website, and will be allocated to either the “parenteral nutrition” or the “oral feeding” group. After randomization, the patient will be given the appropriate patient-information leaflet for the allocated group, after oral information has been provided to the patient.

*Day 1:* The informed consent form, duly signed by the patient and the investigator, will be retrieved by a clinical research assistant. A first consultation will take place at this time to perform standard clinical evaluation and, in particular, to identify and manage any factors that might potentially promote anorexia and digestive disorders. Performance status will be evaluated by using the Karnofsky score, demographic and socioeconomic data will be recorded, and a complete nutritional evaluation, including CRP, albumin, and prealbumin will be performed in all patients. Patients allocated to the parenteral nutrition group will also undergo biological tests before initiation of nutrition. A consultation with a dietitian will be held for all patients. No systematic dietetic follow-up is planned for the study; the cadence of the follow-up consultations is left to the discretion of the relevant healthcare professionals in accordance with their standard practice.

Last, each patient will complete the QLQ-C15-PAL, and patients who are aware of the palliative status of their disease will also complete the QUAL-E.

*From day 1 to day 60:* Patients will have regular follow-up consultations during the follow-up period; at least once per month, based on the patient’s planned attendance at the hospital (for oncology consultations or scheduled admission). The Karnofsky index, quality-of-life evaluation, and details of energy intake will be recorded at each consultation. Nutritional parameters (weight, CRP, prealbumin, albumin) will also be recorded for all patients. Adverse effects will be reported, and serious adverse events will be declared to the relevant pharmacovigilance authorities in the University Hospital of Besancon, France. For patients in the parenteral nutrition group, quantity and duration of nutrition actually administered will be recorded, and complete biological evaluation will be performed (including blood gases, evaluation of phosphorus, magnesium and calcium levels, hepatic enzymes and triglycerides, and evaluation of glycosuria and ketonuria).

Beyond 2 months of treatment, discontinuation or not of parenteral nutrition will be left to the physician’s discretion.

*From day 61 until death*: Quality of life, energy intake, weight, and performance status by using the Karnofsky index will be recorded once per month. Any serious adverse events will be declared to the relevant pharmacovigilance authorities.

### Assignment of interventions

The study will comprise two arms: one intervention group (parenteral nutrition group) and one control group (oral feeding group). The Zelen single-consent design requires that consent be obtained only from the intervention group, for their participation in a study involving initiation of parenteral nutrition. The patient will be informed of the study requirements (inclusion criteria, aims, composition, expected effects and benefits, foreseeable risks, contraindications and so on) to allow them to decide whether to consent to participate. The information delivered to the patient will not mention randomization. Patients allocated to the oral feeding group will be informed about their management, and will consent only to completion of the quality-of-life questionnaire. Patients randomized to the intervention group may refuse to consent to the intervention, in which case, they will be included in the control group.

The distribution of patients between the study arms will be performed by the investigator during the first consultation to verify eligibility criteria, by means of Internet access to a 1-to-1 randomization list, balanced by center, and generated automatically by using SAS software (version 9.3; SAS Institute Inc., Cary, NC, USA) and administered by the Clinical Investigation Centre of the University Hospital of Besancon, France.

#### Sample size and recruitment

In a bilateral situation, a *P* value of <0.0166 will be considered clinically and statistically significant after Bonferroni adjustment for multiple testing, to ensure an overall bilateral alpha risk of 5%. To detect the minimal clinically significant difference (at least 10 points, with a standard deviation of 11 points), with a bilateral alpha risk of 0.0166 and a power of 80%, 54 patients per group are required. Interim analysis to evaluate the primary hypothesis is planned when half the patients have been included and have at least 2 months of follow-up. This interim analysis will be performed according to the rules established by O’Brien and Fleming (Alpha Spending Function with bounds calculated by O’Brien’s method) [24].

The calculation of the sample size takes into account the specificity of the randomization by the Zelen design, and the primary end point of quality of life measured with a multidomain questionnaire:

- Two groups of equal size, constituted by random assignment; one group will receive standard therapy, and the other group will receive the experimental therapy, or standard therapy if the patients refuses to consent to experimental therapy. The heterogeneity of the intervention group will increase the variance of the difference (Δ_z_) between groups for the primary end point, as compared with classic randomization (Δ_c_), albeit without introducing a measurement bias. The increase in variance will increase with the percentage (Φ) of subjects who receive standard treatment in the intervention group, with [25].

In these conditions, the number of subjects must be multiplied by (1/(1 - Φ)^2^). For the ALIM-K study, we estimate that the percentage (Φ) of subjects who receive standard treatment in the intervention group will be low (estimated at around 10% by experts).

- Quality of life, as measured by the questionnaire, will yield a score for each domain. To take into account the multiplicity of comparisons, the Bonferroni correction will be applied to the alpha risk.
- A variation in quality-of-life score of at least 10 points (on a scale of 0 to 100) will be considered as clinically significant.

At an alpha risk of 0.0166, power of 80%, and a percentage Φ of 10%, the number of subjects required to detect a difference of at least 10 points (±11) in quality-of-life score is at least 70 per group (54 × 1.25), or 140 patients in total.

We thus plan to include 16 patients in each of the 10 participating centers ( eight in each group in each center. Participating centres are all specialist cancer institutes, with a high recruitment potential for this type of (relatively frequent) patient profile. Indeed, a preliminary survey performed for the purposes of prior grant applications suggested that more than 20 such cases per year are treated in participating centers. Based on these recruitment estimates with an anticipated moderate inclusion rate, the inclusion period is scheduled to last 30 months, and the total duration of the study including setup, data collection, and analysis will be 42 months.

### Statistical analysis

All analyses will be performed on an intention-to-treat basis. A formal statistical analysis plan will be prepared before the database lock. Any deviation from the statistical analysis plan will be detailed and discussed in the study report.

Clinical and sociodemographic variables collected at baseline will be described as mean ± standard deviation (SD) for normally distributed continuous variables, the median (interquartile) range for nonnormally distributed variables and number (percentage) for qualitative variables. The number of health-related quality-of-life questionnaires completed at each measurement point will be reported.

#### Primary objective

A treatment group will be considered as superior to the other if at least one of the three domains of the primary end point (physical function, overall health status/quality of life, or fatigue) of the QLQ-C15-PAL questionnaire is clinically and statistically significantly better, without any clinically and statistically significant deterioration in at least one of the other two domains.

Comparison of the three domains of the QLQ-C15-PAL questionnaire (between the parenteral nutrition and oral feeding groups) will be performed on an intention-to-treat basis for all patients randomized and included, by using ANOVA on scores at 2 months. Analyses will be adjusted for the presence (or not) of palliative chemotherapy. The variation in the score for each domain between inclusion (day 1) and day 60 will be compared with ANOVA, taking into account stratification variables and potential confounding factors, such as center, type of cancer, and patient characteristics.

#### Change in quality of life over time

Quality-of-life scores will be generated according to algorithms described in the EORTC scoring manual. The mechanism of missing scores will be studied and classified according to the methods of Diggle [26] and Rubin [27] (that is, MCAR (Missing Completely At Random), MAR (Missing At Random), and MNAR (Missing Not At Random). The mechanism of missing data will be determined by comparing patients with no missing scores with those with at least one missing score at baseline, according to baseline characteristics of patients. If required, more detailed patterns will be defined. If missing data are MNAR, multiple imputations may be performed. Otherwise, simple imputation by the mean will be retained.

The time to quality-of-life score deterioration (TTD) will be defined as a modality of longitudinal quality-of-life analysis and will be defined as the time from inclusion in the study to a first deterioration, with a minimum clinically significant difference of at least 5 points as compared with the baseline score, or death of any cause, whichever occurs first [28, 29]. Although three domains of the EORTC QLQ-C15-PAL are being targeted for the primary end point, scores from all domains of the questionnaire will be studied as secondary analyses. The TTD curves will be calculated by using the Kaplan-Meier method and described, by using median and 95% confidence interval (CI). TTD curves will be compared according to treatment group by using the log-rank test and univariate Hazard Ratio with 95% CI. A multivariate Cox regression model will then be performed to investigate potential prognostic factors of TTD. All variables collected at baseline will be tested by univariate analysis. Variables with a *P* value ≤0.20 by univariate analysis will be eligible for inclusion in the multivariate Cox analysis.

Mixed-model analyses of variance for repeated measures will also be performed to test a time effect, a treatment effect, and an interaction between time and treatment. If missing data are not completely at random, a pattern-mixture model will be used.

Analysis of the secondary end points will focus first on comparison of overall survival. Overall survival is defined as the time from study registration to death of any cause. Patients who are lost to follow-up will be counted at the date of their last follow-up.

For all time-to-event end points, the 95% CI of the median survival will be calculated with the Brookmeyer and Crowley method [30]. The two treatment arms will be compared with the log-rank test.

Hazard ratios with 95% CI will be estimated by using Cox’s univariate proportional hazards model. Follow-up will be estimated by using reverse Kaplan estimation.

A multivariate Cox regression model will be performed to investigate potential prognostic factors. All variables collected at baseline will be tested in univariate analysis. Variables with an univariate *P* value ≤0.20 will be eligible for multivariate Cox analysis. A bootstrap procedure will be used to check internal validation of the model. The discriminatory capacity of the model will be assessed with Harrell C statistic. Reclassification ability will be examined with Net Reclassification Improvement (NRI) [31] and Integrated Discrimination Improvement (IDI) [32].

### Ethics

#### Research ethics approval

This protocol is governed by French legislation concerning interventional biomedical research and, as such, was submitted to the local ethics committee (Comité de Protection des Personnes Est II) and approved on January 23, 2012, under the number 11/623. The study was also approved by the French Health Products Safety Agency (Agence Nationale de Sécurité du Médicament et des produits de santé, ANSM) on May 14, 2012. The database was registered with the French national authority for the protection of privacy and personal data (Commission Nationale de l’Informatique et des Libertés, CNIL). Computerised data are processed anonymously. An anonymous identification code will be attributed to each participating in the study, and the list identifying participating patients with their personal data will be stored by the investigator and kept strictly confidential and will not be disclosed to the study sponsor.

To offset any potential remarks on the ethical issues raised by the use of the Zelen design, we came to an agreement with the President of the Ethics Committee that approved the study that we would also consult the Institutional Review Board of the University Hospital of Besancon, as well as a local group of patient associations to obtain their opinion on the choice of randomization method. The study synopsis, a short note explaining the specificities of the Zelen design (accompanied by a figure), and the patient information leaflets from the study were provided. The study was examined by the Institutional Review Board who gave their approval, indicating that the study appeared to protect the patients and was likely the only means to perform a study of this type.

#### Information and consent

Information will be given to all patients on the day of the first consultation. Informed-consent forms will be signed by both the patient and the investigator, with a copy for each, and the original stored by the investigator. Participants are informed in the patient-information leaflet that the data generated by the study will be conserved in electronic format for 15 years. In particular, it is noted that individual data relating to them will be accessible only to the investigator, or to the relevant health authorities for official inspection or a quality audit by official representatives appointed by the sponsor.

Contrary to the classic randomization procedure for clinical trials, the Zelen design proposes that randomization be performed before patients give their consent to participate. The patient information therefore contains no mention of the randomization procedure to minimize the difficulty of obtaining informed consent, thus increasing participation. Once randomization has been performed, patients assigned to the intervention group are then approached for consent (single-consent design). In this design, the patient information and informed consent discuss only the modalities related to the intervention (that is, parenteral nutrition) inclusion criteria, objectives, composition, expected effects, foreseeable risks and benefits, contraindications, administrative issues, follow-up. However, in the ALIM-K study, the patients in both groups will give consent for completion of the quality-of-life questionnaire.

## Discussion

A well-designed, randomized, controlled study comparing parenteral nutrition with oral feeding is needed to reach a consensus on the best form of nutrition in malnourished patients at the palliative phase of advanced cancer. Studies of this type have previously been performed in many patient populations, including patients with cancer [33–37], but with only a very few studies in patients at the palliative phase [12]. This latter study highlights the difficulty of performing randomized controlled trials in palliative care, because the inclusion of the sample size of 309 patients took 6 years.

Performing randomized clinical trials on nutrition in patients at the palliative stage of advanced cancer, a particularly vulnerable population, remains a challenge. It requires the ultimate respect for the patients, many of whom are totally opposed to the idea of any kind of research at this stage of their life and illness. Indeed, being too ill was the most frequently cited motive for refusal to participate in a randomized study by Rabow *et al*. [38] comparing the efficacy of palliative care with standard management in outpatient care for patients with advanced congestive heart failure, chronic obstructive pulmonary disease, or cancer. It is often difficult for patients and their families to accept the idea that research would still be possible at this stage of their illness, let alone the idea that random allocation might be the best option, given the medical uncertainty surrounding the efficacy of the mode of nutrition to be administered. Furthermore, randomization for patients in this context can appear to the patient to “dehumanize” the relation with caregivers and the healthcare system, which can justifiably cause considerable worry and anxiety in the face of the medical community’s uncertainty.

To avoid the possible information bias mentioned and its associated deleterious effects on participation rates, we decided to use the Zelen single-consent design for the ALIM-K study. This choice was approved by the Ethics Committee, the Institutional Review Board, and a group representing patient associations. Many experts initially considered the Zelen method to be a violation of the ethos, if not the very letter of the law, regarding informed consent [39]. However, the method was found to be attractive for research in situations of great precariousness and is used in a variety of different contexts [40]. Patients remain free to express their preference for another treatment approach at any time.

Nonetheless, recruitment remains challenging for several reasons. The methodology of the Zelen design used in the ALIM-K study provides an opportunity to investigate the factors that influence the decision by patients or physicians to accept (or not) artificial nutrition in the setting of palliative care for advanced care. At the palliative stage of cancer, the main end point is quality of life, even though this term may appear to be a misnomer to many patients at the end of life. The QLQ-C15 PAL questionnaire used in our study presents the advantage of addressing simple issues related to daily life, and is quick and easy to complete.

Because our randomization procedure takes into consideration the fragility of this patient population, and the design is close to the reality of routine practice, it will be interesting to see which factors emerge as predictors of refusal or acceptance of artificial nutrition.

## Trial status

Between May 2012, and April 2013, 13 patients were included in two centers to check the feasibility and understanding of the questionnaires, as well as their acceptability. Qualitative analyses of these pretest data confirmed that methods and questionnaires are acceptable and that the study is feasible. The main adjustment to result from this test phase was greater precision required in some of the inclusion criteria.

At the time of submission of the manuscript, the ALIM-K study is ongoing. All 10 centers were enrolling progressively, and the last participating center (Lille) was opened on April 16, 2014. Patient recruitment is not complete, at the time of submission.

## Abbreviations

ANOVA: analysis of variance
ANSM: Agence Nationale de Sécurité du Médicament et des produits de santé (French Agency for the Safety of Health Products)
BMI: body mass index
CI: confidence interval
CNIL: Commission Nationale de l’Informatique et des Libertés (French National Authority for the Protection of Privacy and Personal Data)
CRP: C-reactive protein
EORTC: European Organisation for Research and Treatment of Cancer
ESPEN: European Society for Parenteral and Enteral Nutrition
IDI: Integrated Discrimination Improvement method
IRB: institutional review board
MAR: missing at random
MCAR: missing completely at random
MNAR: missing not at random
NRI: Net Reclassification Improvement method
QLQ-C15 PAL: Quality of Life Questionnaire Core 15 Palliative
QLQ-C30: Quality of Life Questionnaire Core 30
QUAL-E: Questionnaire of Quality of Life at End of Life
SD: standard deviation
SFNEP: Société Francophone Nutrition Clinique et Métabolisme
TTD: time to quality of life score deterioration.
